# The temporal dynamics of chromosome instability in ovarian cancer cell lines and primary patient samples

**DOI:** 10.1371/journal.pgen.1006707

**Published:** 2017-04-04

**Authors:** Signe Penner-Goeke, Zelda Lichtensztejn, Megan Neufeld, Jennifer L. Ali, Alon D. Altman, Mark W. Nachtigal, Kirk J. McManus

**Affiliations:** 1 Department of Biochemistry and Medical Genetics, University of Manitoba, Winnipeg, Manitoba, Canada; 2 Research Institute in Oncology and Hematology, CancerCare Manitoba, Winnipeg, Manitoba, Canada; 3 Department of Obstetrics, Gynecology and Reproductive Sciences, University of Manitoba, Winnipeg, Manitoba, Canada; Cleveland Clinic Genomic Medicine Institute, UNITED STATES

## Abstract

Epithelial ovarian cancer (EOC) is the most prevalent form of ovarian cancer and has the highest mortality rate. Novel insight into EOC is required to minimize the morbidity and mortality rates caused by recurrent, drug resistant disease. Although numerous studies have evaluated genome instability in EOC, none have addressed the putative role chromosome instability (CIN) has in disease progression and drug resistance. CIN is defined as an increase in the rate at which whole chromosomes or large parts thereof are gained or lost, and can only be evaluated using approaches capable of characterizing genetic or chromosomal heterogeneity within populations of cells. Although CIN is associated with numerous cancer types, its prevalence and dynamics in EOC is unknown. In this study, we assessed CIN within serial samples collected from the ascites of five EOC patients, and in two well-established ovarian cancer cell models of drug resistance (PEO1/4 and A2780s/cp). We quantified and compared CIN (as measured by nuclear areas and CIN Score (CS) values) within and between serial samples to glean insight into the association and dynamics of CIN within EOC, with a particular focus on resistant and recurrent disease. Using quantitative, single cell analyses we determined that CIN is associated with every sample evaluated and further show that many EOC samples exhibit a large degree of nuclear size and CS value heterogeneity. We also show that CIN is dynamic and generally increases within resistant disease. Finally, we show that both drug resistance models (PEO1/4 and A2780s/cp) exhibit heterogeneity, albeit to a much lesser extent. Surprisingly, the two cell line models exhibit remarkably similar levels of CIN, as the nuclear areas and CS values are largely overlapping between the corresponding paired lines. Accordingly, these data suggest CIN may represent a novel biomarker capable of monitoring changes in EOC progression associated with drug resistance.

## Author summary

Ovarian cancer is one of the most lethal cancers in women due to the high prevalence of drug resistant disease. New insight into the biology causing drug resistance is required to reduce death rates associated with the disease. In many cancer types, chromosome instability (CIN; or abnormal numbers of chromosomes) is associated with aggressive tumours, the acquisition of multi-drug resistance and poor patient outcome, yet CIN is poorly characterized in ovarian cancer. Here, we employ a new microscopy-based approach to examine the presence and dynamics of CIN within single cells obtained from ovarian cancer patients collected over time. We show that CIN is associated with every sample and further show that increases in CIN are associated with treatment and/or the development of drug (platinum) resistant disease. Although additional studies are required, these findings suggest CIN may be a new biomarker that can monitor disease progression, particularly in response to therapy. Finally, we demonstrate that CIN is low in two cellular models of drug resistant disease. Collectively, our findings strengthen the need for studies employing patient-derived samples to accurately assess the abnormal biology associated with the disease, and cautions the use of these cell models in studies related to drug resistance that may arise from CIN.

## Introduction

Novel insight into epithelial ovarian cancer (EOC) pathogenesis is essential to reduce the high mortality rate caused by recurrent, drug-resistant disease. EOCs comprise the most frequent type of ovarian cancer (incidence of 10.8 per 100 000 women in Canada) and have the highest mortality rate (152 000 deaths worldwide in 2012)[1]. There are two histologic subgroups where Type 1 includes mucinous, low grade serous, low/moderate grade endometrioid, and clear cell cancers, and Type 2 includes moderate/high grade serous, high grade endometrioid, undifferentiated, and malignant mixed mesodermal tumours[2]. Chemotherapy may be the only viable treatment in EOC patients where the mass or spread of the tumours limits surgical debulking. While many patients may respond initially to adjuvant chemotherapy (usually 6–9 cycles of cisplatin or carboplatin, plus a taxane), as much as 75% of patients will relapse within 18 months[3] with chemoresistant disease. Chemoresistant disease is defined as disease recurrence within 6 months of initial treatment. EOCs that reoccur are typically incurable; however, response rates have been observed in 10–30% of patients treated with gemcitabine, liposomal doxorubicin (Caelyx), or topotecan, which has increased their progression-free survival[4–6]. One of the fundamental priorities identified at the 2015 Ovarian Cancer Action meeting was to characterize genome instability in acquired drug resistant disease[7]. We propose that a more complete understanding of the molecular mechanisms driving genomic instability in EOC is essential to enhance our prognostic, diagnostic, and therapeutic capabilities.

In general, chromosome instability (CIN) is one of several mechanisms suspected to drive and promote genome instability[8–10]. CIN is defined as an increase in the rate at which whole chromosomes or large parts thereof are gained or lost (see [11]). To accurately assess CIN mandates the use of single cell approaches capable of characterizing genetic or chromosomal heterogeneity within a given population[11]. CIN typically manifests due to errors in DNA replication, DNA repair or chromosome segregation (reviewed in [12, 13]), and is of particular interest as in many cancer types, CIN is associated with aggressive tumors, the acquisition of drug resistance and poor patient prognosis[14–19]. Numerous studies have evaluated various aspects of genome instability in EOC, with a particular focus on the most prevalent form of EOC, high-grade serous ovarian cancer (HGSOC)[20–29]. For example, HGSOC is characterized by gene copy number variations, structural variants, single nucleotide variants, and sometimes highly rearranged genomes[23, 27, 30]. However, many of these studies employ DNA sequencing or single nucleotide polymorphisms (SNP) analyses to characterize this genome instability in DNA extracted from large numbers of cells (e.g. 10^3^ to 10^6^ cells)[20–29]. Thus, these results are heavily impacted by population averaging and are incapable of characterizing the karyotypic complexity and heterogeneity typically associated with CIN. Furthermore, because aneuploidy (*i*.*e*. abnormal chromosome numbers) is frequently used as a surrogate marker for CIN, traditional studies have also employed cytogenetic approaches including karyotypic analyses and comparative genomic hybridization (CGH) to assess aneuploidy or CIN within a given sample. While these approaches have identified karyotypic differences between samples, they typically only report a modal karyotype for any given sample, which is usually drawn from 25–30 mitotic chromosome spreads. Consequently, these approaches are limited in scope as they are costly, time consuming, limited in capacity, and incapable of adequately quantifying the cell-to-cell heterogeneity induced by CIN within hundreds of cells from a given sample, particularly over time.

To begin to address the limitations of conventional approaches and more accurately quantify the heterogeneous nature of CIN, we previously developed and validated two independent image-based approaches that evaluate surrogate markers of CIN, namely nuclear areas[31] and CIN score (CS) values[32]. To better characterize CIN, we have now combined these complementary, quantitative approaches and applied them in the assessment of CIN from various EOC samples representing various disease states collected over time. Briefly, changes in nuclear areas typically correlate with large-scale alterations in DNA content (*i*.*e*. ploidy), while a CS is a metric devised to describe gains and losses of three specific chromosomes within a sample that can be associated with both small- and large-scale gains. CS values are determined using fluorescence *in situ* hybridization (FISH) and chromosome enumeration probes (CEPs) for specific centromeres (*e*.*g*. chromosomes 8, 11 and 17), to assess their presence within samples. Because centromeres are essential chromosome elements, the number of FISH signals (foci) present within a nucleus is indicative of chromosome copy numbers. Accordingly, deviations from two FISH signals/chromosome/cell identify aneuploid cells, or those exhibiting CIN[32]. Thus, unlike the traditional approaches, this novel single cell approach is uniquely capable of quantifying CIN in hundreds of cells both within and between samples over time.

In this study, we employed quantitative imaging microscopy to evaluate CIN (i.e. nuclear areas and CS values) within serial samples isolated from the ascites of EOC patients and within two established EOC cell line models of platinum resistance (PEO1 and PEO4; A2780s and A2780cp). Here we show the patient samples generally harbor increased levels of CIN as reflected by large changes in nuclear areas and CS values. Interestingly, these levels can vary significantly between serial samples indicating aneuploidy and CIN are dynamic in EOC patients and appear to reflect changes in the underlying biology associated with disease status, including responsive, resistant and recurrent disease. By contrast, we show the platinum resistant cell lines exhibit similar levels of CIN, as demonstrated by similar nuclear areas and CS values, suggesting they harbor similar levels of CIN. Collectively, these data suggest that nuclear area and CS values may be useful biomarkers capable of tracking and monitoring disease response and resistance.

## Results

### CIN is associated with EOC patients samples

**Fig 1:**
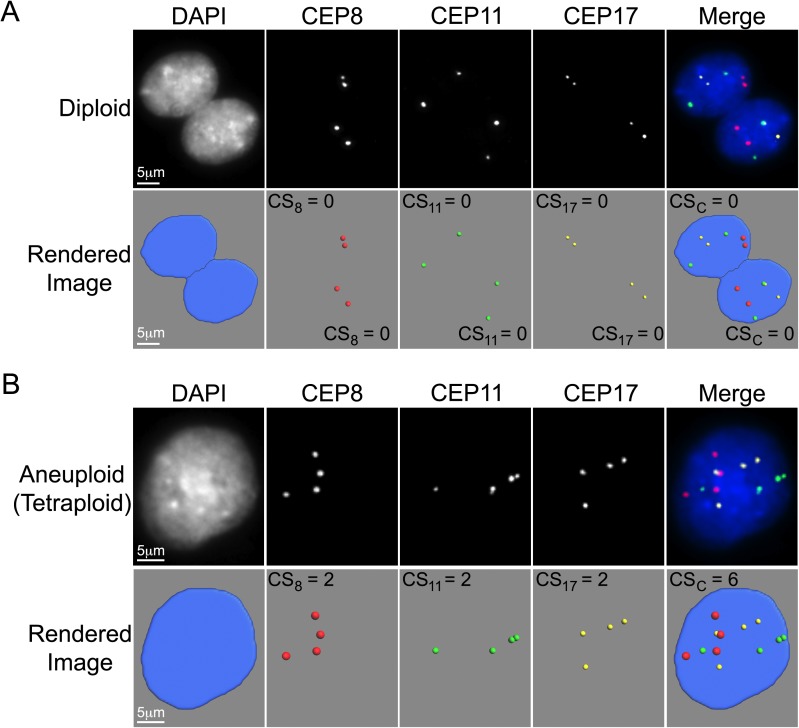
Images Depicting Diploid and Aneuploid States within Primary EOC Cells. **(A)** Representative high resolution images presenting two diploid EOC nuclei counterstained with DAPI (blue) and labeled with CEPs 8 (red), 11 (green) and 17 (yellow). Presented are the widefield fluorescence images (top) and the corresponding rendered images (bottom) generated and employed to assess both nuclear areas and CS values. Note that within each nucleus, two foci per chromosome evaluated are present indicating the cells are diploid for chromosomes 8, 11 and 17. For illustrative purposes, the individual (CS_8_, CS_11_ and CS_17_) and combined CS (CS_C_) values have been included within each rendered image. **(B)** Representative high resolution, widefield fluorescence images (top) and the corresponding rendered images (bottom) of an aneuploid nucleus. Note that four foci are present for each CEP evaluated indicating the cell is tetraploid for chromosomes 8, 11 and 17. For illustrative purposes, the CS values have been included within each rendered image.

**Fig 2:**
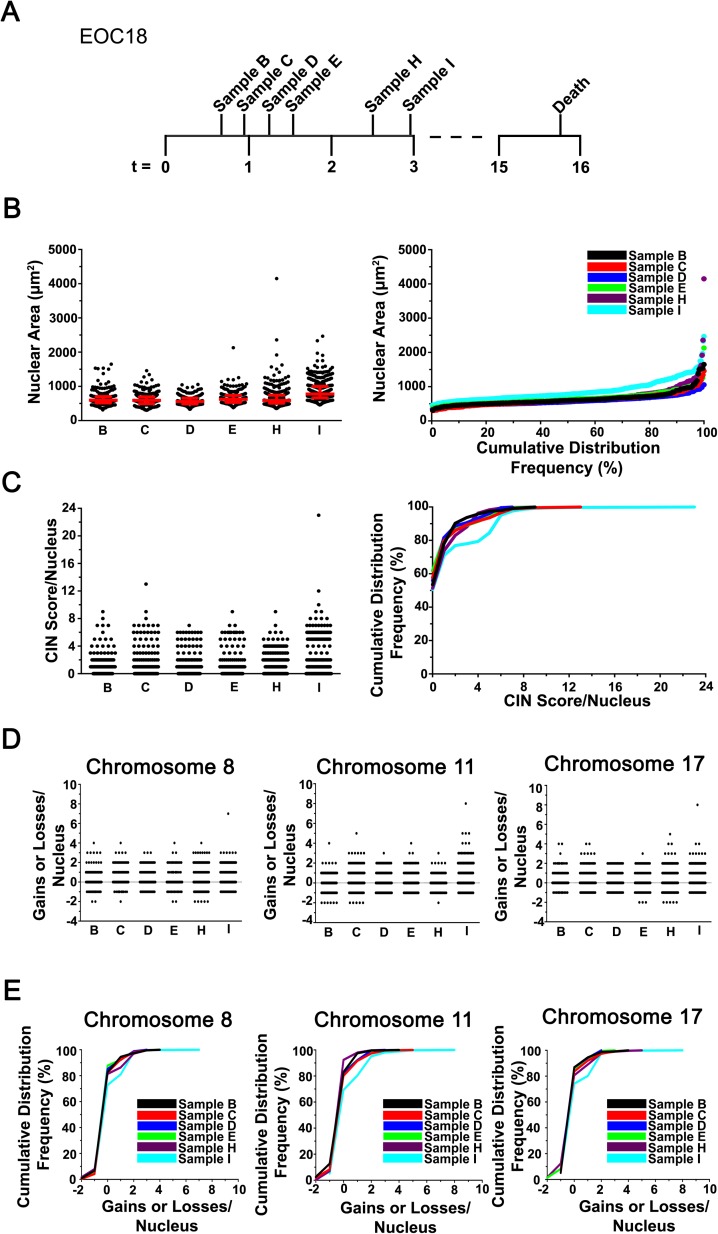
CIN is Associated with Primary EOC. **(A)** Timeline (months) presenting the six collection times (samples B, C, D, E, H and I) from patient EOC18 (who declined treatment options) prior to death. **(B)** Scatter plot (left) presenting the nuclear area distributions (black circles) and the associated interquartile ranges (25^th^, 50^th^ and 75^th^ percentiles; red horizontal lines). Cumulative distribution frequency graph (right) depicting nuclear areas arranged from smallest to largest. **(C)** A scatter plot (left) presenting the CS_C_ distributions of samples B to I, and the corresponding cumulative CS_C_ distribution frequencies (right). Note that the absolute values of gains and losses are presented for the combined CS values (CS_C_; CEPs 8, 11 and 17), and that a CS_C_ value of 0 identifies a diploid nucleus for each chromosome evaluated (*i*.*e*. 2 copies of CEPs 8, 11 and 17). (**D)** Scatter plots presenting the gains and losses in CS_8_ (left), CS_11_ (middle), and CS_17_ (right) for each nucleus analyzed within each sample. Note that both gains (positive values) and losses (negative values) are shown for each individual CEP evaluated. **(E)** Cumulative distribution frequency graphs for CS_8_ (left), CS_11_ (middle) and CS_17_ (right).

To determine whether CIN was associated with EOC, we employed our recently established approaches[31, 32] and assessed CIN using primary cancer cell samples isolated from patient ascites (S1 Table). Production of ascites is dependent on the individual patient, and its production often changes throughout the course of the disease and with treatment response[33, 34]. Primary cell samples were labeled with CEPs 8, 11 and 17, counterstained with DAPI and subjected to quantitative imaging microscopy, where both nuclear areas (DAPI) and CS values were determined for hundreds of cells per sample (see Materials & Methods) (Fig 1). Six serial samples were isolated and analyzed from EOC18, who was diagnosed with ovarian adenocarcinoma and refused chemotherapy (Fig 2A). In general, there was a striking degree of heterogeneity observed for nuclear areas within each of the samples (Fig 2B). The minimum total range observed was 726μm^2^ (sample D), while the maximum range was 2794μm^2^ (sample H). In addition, the mean nuclear areas were similar within the first five samples (B, 640μm^2^; C, 615μm^2^; D, 584μm^2^; E, 663μm^2^; H, 676μm^2^), but increased 1.35-fold within the final sample (I, 866μm^2^) (S2 Table). Similarly, the interquartile ranges (Fig 2B, left), which reflect the heterogeneity within a population were also largely overlapping within the first five samples, and increased within the sixth (S2 Table). Fig 2B (right) shows that the distribution of sample I was both visually and statistically (S3 Table) distinct from all other samples. Thus, these data show there is considerable nuclear area heterogeneity within EOC18 that increases with time and disease progression.

Next, CS values were compared between samples from EOC18 (see Materials & Methods). Briefly, a CS describes both the gains and losses of fluorescent CEP foci within diploid nuclei from a given sample. Under normal conditions, a diploid nucleus harbors two foci per CEP (Fig 1A), while aneuploid cells will deviate (Fig 1B; S1 Fig). By definition, CS values reveal deviations from the diploid state (operationally set to 0) as diploid cells have CS values = 0, whereas aneuploid cells have CS values that deviate from 0 (Fig 1; S1 Fig). FISH was performed, and in agreement with the nuclear areas detailed above, the overall CS_C_ distribution ranges (Fig 2C, left) were largely overlapping within the first five samples (B to H) (CS_C_ values typically ≤ 8) and were largest within the final sample (maximum CS_C_ value = 24). Two sample Kolmogorov-Smirnov (KS) tests comparing the CS_C_ distributions revealed that all pairwise combinations involving sample I were statistically different (*p*-value <0.05), while all other pairs were not significant (S4 Table). Similar trends were also observed for the individual CS values (Fig 2D and 2E), as KS-tests revealed that all but one of the CS_8_, CS_11_ or CS_17_ pairwise combinations involving sample I were significant (S4 Table). Collectively, these data show that CIN (as measured by nuclear areas and CS values) is present in all EOC18 samples, and further show that CIN remains relatively stable within the first five samples (B to H; ~2 months), but begins to increase within the final sample (I).

### CIN is dynamic following carboplatin and paclitaxel treatments

**Fig 3:**
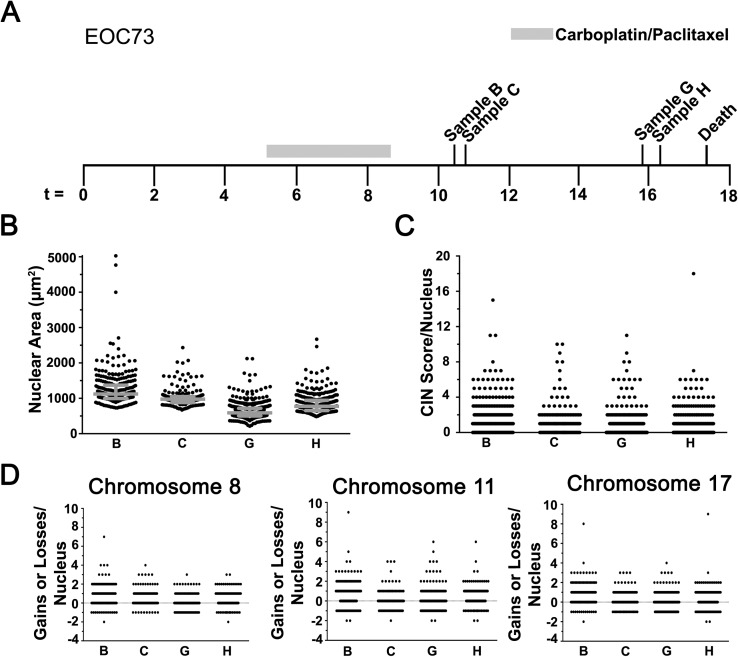
CIN is Dynamic and Changes in Response to Frontline EOC Chemotherapy. **(A)** A timeline (months) presenting the collection times for samples B, C, G and H from EOC73 relative to Carboplatin/Paclitaxel treatment. **(B)** Scatter plot depicting the nuclear area distribution for each sample with the interquartile ranges indicated in grey. **(C)** Scatter plot depicting the CS_C_ values for each nucleus evaluated within the indicated samples. **(D)** Scatter plots presenting the gains and losses in CS_8_ (left), CS_11_ (middle) and CS_17_ (right) for each nucleus analyzed within sample.

The above observations suggest changes in CIN may reflect alterations in the underlying biology associated with disease progression. To assess whether CIN exhibits distinct temporal dynamics after the patient has received chemotherapy, CIN was evaluated in EOC73, a patient with an ovarian carcinosarcoma whose samples were collected post-carboplatin/paclitaxel treatment (Fig 3A). As predicted, nuclear area analyses revealed large and dynamic total distribution ranges and mean nuclear areas within each sample (Fig 3B; S2 Table). This is further illustrated in S2A Fig, which shows largely non-overlapping and statistically distinct cumulative nuclear area distribution frequencies for each sample (S5 Table). CS_C_ values were also evaluated and generally show distinct distribution profiles within each sample (Fig 3C). Large overall ranges were observed in each sample (CS_C_ values typically ≤ 8) indicating a high prevalence of aberrant numbers of chromosomes 8, 11 and 17. The cumulative CS_C_ distribution frequencies (S2B Fig) mirror the nuclear area data, and with the exception of samples C and H, all remaining pairwise comparisons are statistically distinct (S6 Table). As expected, similar, albeit less distinct trends were also observed for the individual CS values (Fig 3D; S2C Fig). KS-tests reveal that CS_8_ values were the most different between samples, while CS_11_ and CS_17_ values were most similar (S6 Table). Collectively, the above data show CIN is dynamic following carboplatin/paclitaxel treatments and initially decreases (sample B to G) before increasing (sample H), implying changes in CIN reflect the temporal therapeutic effects and tumor response to chemotherapy.

### CIN increases in resistant recurrent disease

**Fig 4:**
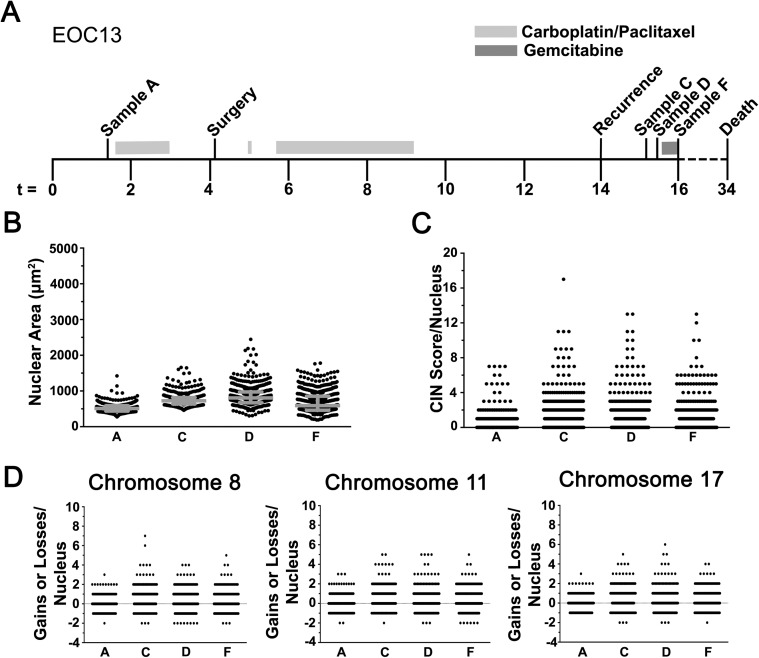
CIN Increases in Recurrent Disease. **(A)** Timeline (months) indicating the time of collection for samples A, C, D and F from patient EOC13 relative to treatments, surgery and recurrence. **(B)** Scatter plot depicting the nuclear area distributions and interquartile ranges (grey). **(C)** Scatter plot presenting the overall distribution of CS_C_ values within each sample. **(D)** Scatter plots for each of the individual CS values; CS_8_ (left), CS_11_ (middle) and CS_17_ (right).

**Fig 5:**
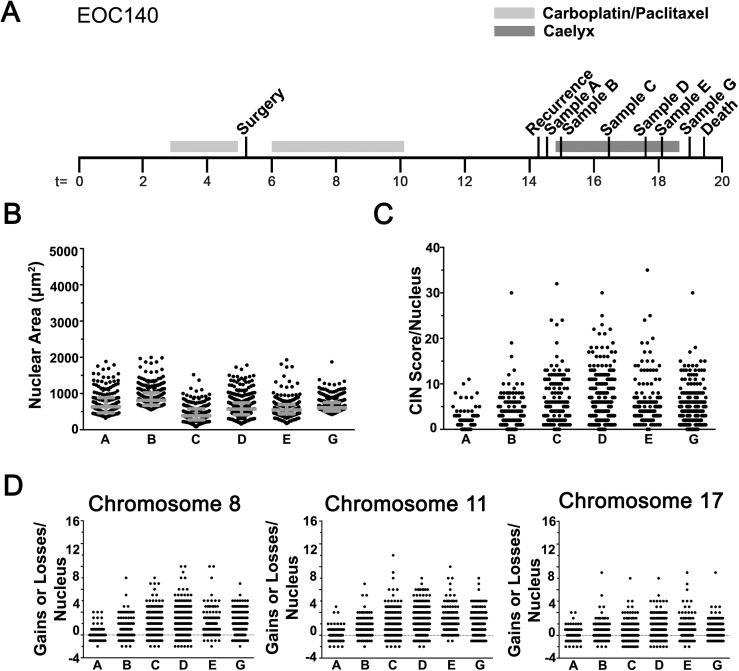
Extensive Levels of CIN are Associated with Recurrent Disease. **(A)** Timeline (months) presenting the collection times of the six samples collected from patient EOC140 relative to surgery, disease recurrence and death. **(B)** Scatter plot presenting the overall distribution of nuclear areas from each sample with the interquartile range indicated in grey. **(C)** Scatter plot of CS_C_ values. Note the expanded range for the overall distributions. **(D)** Scatter plots presenting CS_8_ (left), CS_11_ (middle) and CS_17_ (right) for each nucleus evaluated.

The preceding sections suggest CIN may be a novel biomarker that reflects changes associated with disease progression, recurrence, and/or drug treatments. To assess this possibility, CIN was evaluated in samples isolated from EOC13 (Fig 4A) and EOC140 (Fig 5A), patients diagnosed with recurrent, platinum-resistant HGSOC. Nuclear areas were assessed as above (S2 Table), and visually distinct differences in the distribution ranges were apparent for both patients (Figs 4B and 5B). EOC13 exhibited an overall increase in the total distribution range from sample A to D that decreased in sample F. Statistically significant increases in mean nuclear areas were also observed in samples C and D (752μm^2^ and 849μm^2^, respectively) relative to sample A (520μm^2^), that was followed by a statistically significant decrease within sample F (677μm^2^). Despite the decrease in mean nuclear area within sample F, the interquartile ranges continued to expand and was largest in sample F (Fig 4B). Finally, KS-tests comparing the cumulative nuclear area distribution frequencies (see S3A Fig) reveal all pairwise combinations were statistically distinct (S7 Table). For EOC140, Fig 5B shows that the overall nuclear areas and interquartile ranges were large, generally distinct between samples, and were typically associated with increases in mean nuclear areas apart from samples B to C and D to E (S2 Table). Further, all pairwise combinations evaluating the cumulative nuclear area distribution frequencies (S4A Fig) were statistically distinct (S8 Table) with the exception of samples D and E.

Next, CS values were evaluated and large CS_C_ ranges were observed within each EOC13 sample (Fig 4C; CS_C_ values typically ≤ 8) that subsequent KS-tests revealed were statistically significant (S3 Fig and S9 Table). Similar trends occurred for CS_8_, CS_11_, and CS_17_ (Fig 4D), and KS-tests revealed that most pairwise comparisons were statistically different with the exception of A versus F for both CS_8_ and CS_17_, and D versus F for CS_17_ (S9 Table). Similarly, the CS_C_ values were also dynamic in EOC140 (Fig 5C), however they were generally larger (typically ≤ 20) with maximal values ranging from 30 and 35 in samples B to G, indicating high levels of aneuploidy and CIN. This trend was also observed for the cumulative distribution frequencies (S4B Fig) and was further highlighted by the significant KS-tests for all pairwise comparisons with the exception of samples C vs. D and D vs. E (S10 Table). In agreement with the CS_C_ values, CS_8_, CS_11_ and CS_17_ values exhibit similar dynamic patterns (Fig 5D) with largely distinct distribution patterns observed between samples (S4C Fig; S10 Table). The apparent difference observed between nuclear areas and CS values for EOC140 may suggest that while many chromosomes are dynamically gained or lost, overall there may be selective pressures that maintain or promote selective gains in chromosomes 8, 11 and 17 (Fig 5D). Taken together, the above data show that CIN is prevalent and highly dynamic within EOC140. They further show that high levels of aneuploidy and CIN are present within recurrent disease and that further treatments (*i*.*e*. Caelyx) did not impact the overall size of nuclei nor the CS values within the bulk of the cells evaluated within the samples. These data again show that CIN is dynamically associated with EOC, further supporting its potential utility as putative biomarker.

### Increases in CIN are associated with the development of platinum resistant EOC

**Fig 6:**
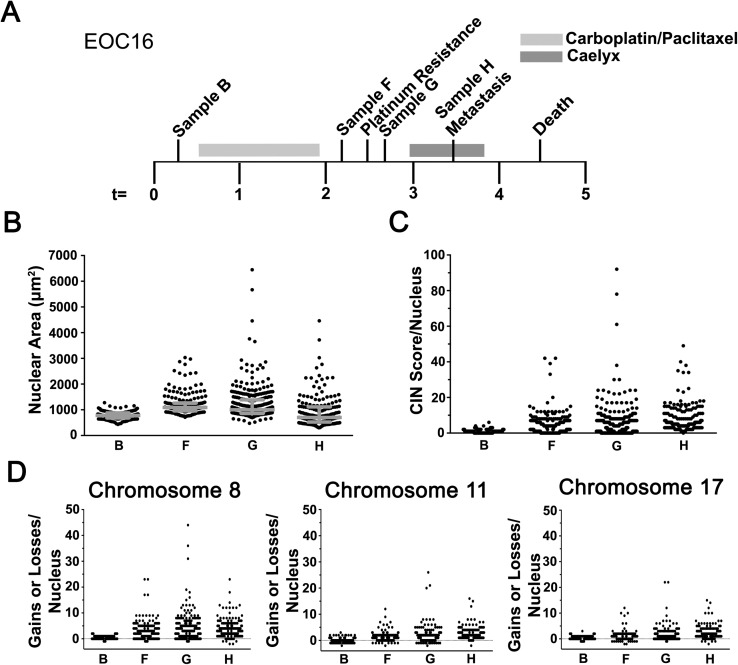
The Levels of CIN Increase in Aggressive and Platinum Resistant Disease. **(A)** Timeline (months) indicating the collection times for samples B, F, G and H from patient EOC16 relative to treatments, platinum resistance, metastasis and death. **(B)** Scatter plot for nuclear areas from the indicated samples (interquartile ranges indicated in grey). **(C)** Scatter plot for the CS_C_ values. (**D)** Scatter plots presenting the overall distribution of CS_8_ (left), CS_11_ (middle) and CS_17_ (right) from each nucleus evaluated within the indicated samples.

CIN is associated with the acquisition of multi-drug resistance in numerous cancer types[14–17], but there is a paucity of data regarding its association with platinum resistant EOC. The findings of the preceding section show CIN is dynamic, and predict high levels of CIN will be associated with platinum resistance (*e*.*g*. EOC140). To explore this possibility, CIN was evaluated in samples from EOC16 (Fig 6A), a patient with platinum resistant disease who was administered Caelyx prior to death. Similar to the platinum resistant samples analyzed above, increases in nuclear area were associated with the acquisition of resistance, or given the rapid progression of the disease, enrichment of platinum resistant cells (Fig 6B). More specifically, increases in the interquartile ranges were observed in samples F and G relative to sample B that were accompanied with significant increases in mean nuclear areas (S2 Table). Surprisingly, sample H (undergoing Caelyx treatment) maintained a large interquartile range despite showing a significant decrease in mean nuclear area relative to sample G (prior to Caelyx treatment). The cumulative nuclear area distribution frequencies (S5A Fig) of the three post-treatment samples appeared similar and distinct from the pre-treatment sample (B), which KS-tests showed were statistically distinct (S11 Table).

In general, the CS_C_ values closely parallel those of the nuclear areas (Fig 6C; S5B Fig) as the CS_C_ values were generally larger (typically ≤ 20) than those of all EOC patient samples evaluated thus far (typically ≤ 8), and were as high as 45 to 93 in samples F, G and H indicating extreme levels of aneuploidy and CIN (S1 Fig). Similarly, the individual CS values exhibited dynamic patterns over time (Fig 6D) as the CS_8_, CS_11_ and CS_17_ values increased from sample B to G and decreased marginally within sample H. Further, CS_8_, CS_11_ and CS_17_ values were maximal in sample G and decreased within sample H, but still exhibited the greatest levels of aneuploidy as indicated by the right-shifted cumulative distribution curve (S5C Fig). Subsequent KS-tests revealed all pairwise combinations were statistically distinct with the exception of sample F versus G for both CS_8_ and CS_17_ (S12 Table). Collectively, these data confirm CIN is both prevalent and dynamic in HGSOC, and further show high levels of CIN are associated with platinum resistance and that Caelyx treatments do not appear to significantly impact nuclear size nor CS values in EOC16.

### Cellular models of drug resistance in EOC exhibit similar levels of CIN

**Fig 7:**
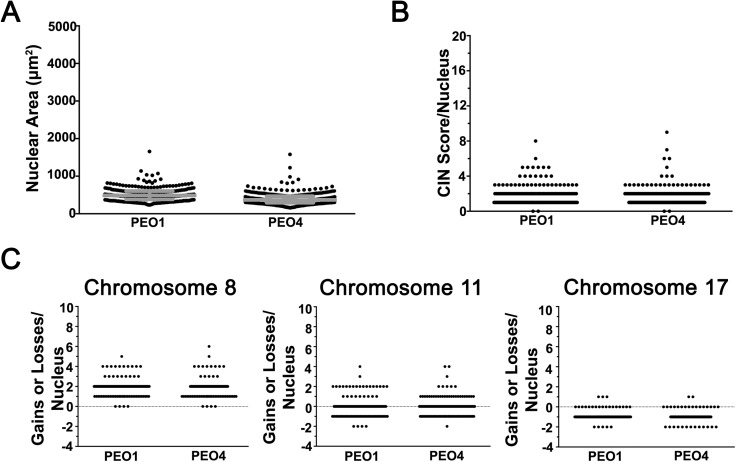
PEO1 and PEO4 Cells Harbor Similar Levels of CIN. **(A)** Scatter plot depicting the nuclear area distribution for PEO1 (sensitive) and PEO4 (resistant) cells with the interquartile ranges (25^th^, 50^th^ and 75^th^ percentiles) identified in grey. **(B)** Scatter plot depicting the CS_C_ distribution for nuclei in PEO1 and PEO4 cells. **(C)** Scatter plots presenting the gains and losses of CEP 8 (CS_8_; left), 11 (CS_11_; middle) and 17 (CS_17_; right) for each nucleus analyzed in PEO1 and PEO4.

We next sought to determine whether CIN was also associated and dynamic in two paired EOC models of drug resistance, PEO1/PEO4 and A2780s/A2780cp. PEO1 (sensitive) and PEO4 (resistant) cells were isolated and derived from a single EOC patient with HGSOC pre- and post-platinum resistance[35], while A2780cp (resistant) cells were derived through dose escalation in the cisplatin sensitive parental line (A2780s) isolated from a patient with ovarian clear cell carcinoma[36]. Nuclear areas were quantified as above and in stark contrast to the patient samples, PEO1/PEO4 cells exhibited relatively small overall distribution and interquartile ranges (Fig 7A; S13 Table). In fact, there was a small ~1.3-fold decrease in mean nuclear area within PEO4 cells (391μm^2^) compared to PEO1 (510μm^2^) along with a significant decrease in the cumulative distribution frequency (KS-test; *p*-value < 0.0001) (S6A Fig). Similarly, the overall and interquartile ranges were small within A2780s and A2780cp cells (S7A Fig; S13 Table), however there was a modest, albeit statistically significant, increase in cumulative nuclear area distribution frequency (KS-test; *p*-value = 0.001) within A2780s relative to A2780cp (S7A Fig). Next, CS_C_ values were calculated and revealed small overall distribution ranges that were typically ≤ 4 (Fig 7B; S7B Fig). In addition, the cumulative distribution frequencies were largely superimposable and not statistically distinct for either PEO1/PEO4 (*p*-value < 0.88) or A2780s/A2780cp (*p*-value = 0.10). Similarly, the CS_8_, CS_11_ and CS_17_ distribution ranges were also largely overlapping (Fig 7C; S7C Fig) as were the cumulative distribution frequencies (S6C Fig; S7D Fig). Collectively, these data show that the paired cell line models exhibit small, yet similar nuclear area distributions and CS values, indicating that the sensitive and resistant cell lines exhibit similar levels of CIN.

### Mean CS values are dynamic and high within aggressive disease

**Fig 8:**
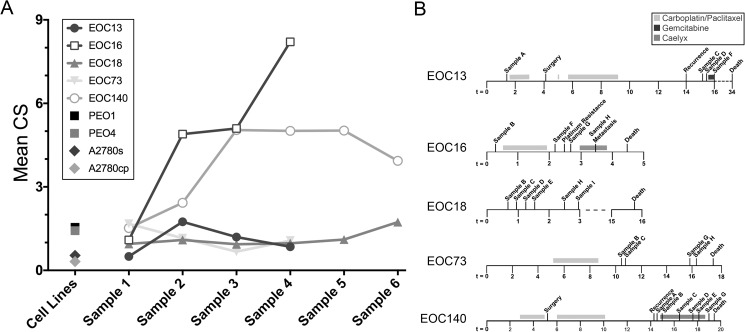
Mean CS Values in Primary EOC Patient Samples and Cell Line Models. **(A)** Graph presenting the mCS values calculated for each patient and sample isolated from each patient, and those of the paired EOC cell line models (PEO1/PEO4 and A2780s/A2780cp). Note that the mCS value in each of the initial patient samples is <2 and remains low in all subsequent samples with the exception of EOC16 and EOC140, which exhibit striking increases in mCS values prior to succumbing to the disease. Also, note that the carboplatin resistant cell lines (PEO4 and A2780cp) both exhibit small decreases in mCS values relative to their sensitive counterparts. **(B)** Timelines presenting the sample collection times for each EOC patient presented in **(A)** relative to disease progression and treatment(s).

To compare CIN between EOC patient samples and the paired drug resistant lines, mean CS (mCS) values were calculated[32]. For reference purposes, a mCS = 0 is indicative of a diploid population, whereas a mCS value >0 identifies an aneuploid population. As shown in Fig 8, the mCS values calculated for each of the initial EOC patient samples was <2, and ranged from 0.499 (EOC13) to 1.675 (EOC73). Interestingly, the mCS value remained relatively static in subsequent samples evaluated from EOC13, EOC16, and EOC140, while those in EOC73 and EOC18 were dynamic. More specifically, the mCS value from EOC18 (refused treatment) were stable for the first four samples before increasing within the final two samples (1.1 and 1.7). Conversely, the mCS values of EOC16 and EOC140 increased dramatically in the second and third samples, respectively, and remained high (mCS >4) in all subsequent samples. The above data predict the drug resistant cell lines, PEO4 and A2780cp will have elevated mCS values relative to the paired sensitive lines. Interestingly, although the sensitive lines have mCS values (PEO1 = 1.547; A2780s = 0.538) that are comparable to those of the initial EOC patient samples, the mCS values are lower in the corresponding drug resistant lines (PEO4 = 1.426; A2780cp = 0.316). Overall, the above results show that mCS values remain relatively low (mCS <2) in stable and responsive disease, but increase dramatically within resistant or aggressive disease suggesting mCS values are a novel candidate biomarker capable of monitoring disease progression and response.

## Discussion

To glean unprecedented insight into the prevalence and dynamics of CIN in EOC patient samples and two ovarian cancer cell models of drug resistance, we combined our recently developed analyses[31, 32] and assessed two surrogate markers of CIN. Using quantitative, single cell imaging approaches we show CIN, as characterized by changes in nuclear areas and CS values, is associated with all EOC samples, and further show it is dynamic and increases in resistant, recurrent disease. A constraint of the current study is that it is drawn from small patient cohorts, which limits the power of the study to draw firm conclusions about the temporal dynamics of CIN until additional samples can be acquired and analyzed. Nevertheless, the results of the current study suggest that alterations in CIN may reflect the underlying genetic changes driving disease progression and drug response. For example, significant differences in mean nuclear areas and CS values occurred in serial samples collected from EOC patients, particularly those undergoing chemotherapy. Results from EOC73 (Fig 3) demonstrate CIN decreases following the initial carboplatin/paclitaxel treatments before increasing within the final sample. Similarly, CIN increases prior to, and immediately following, platinum resistance in EOC16 (Fig 6), but decreases slightly following Caelyx treatments. These data reveal a potential association between platinum resistance and increases in CIN (*i*.*e*. increased heterogeneity). In support of this possibility, CIN remains relatively stable in EOC18 (Fig 2), an individual who refused all treatments. In fact, nuclear areas and CS values remained relatively constant within the first five samples, but increased within the final sample as the disease progressed. Collectively, these observations raise the question of whether changes in CIN reflect the pathologic changes driving disease progression, or those induced by drug treatments.

In agreement with these findings, both cellular models of drug resistance exhibit CIN; however, both models show remarkable similarity between the paired sensitive (PEO1 and A2780s) and resistant lines (PEO4 and A2780cp). Despite PEO4 being isolated 10 months after relapse and being platinum resistant[37], we did not observe elevated levels of CIN (*e*.*g*. nuclear areas and CS values) relative to PEO1. In fact, our data show that PEO1 and PEO4 are similar with respect to nuclear areas and CS values (Fig 7). Although our approach is incapable of detecting segmental aneuploidy (*i*.*e*. gains or losses in portions of chromosomes), our data indicate that chromosomes 8 and 17 are preferentially gained and lost in PEO1 and PEO4, respectively (Fig 7C). Thus, our data show that although PEO1 and PEO4 share many features of CIN, the PEO4 cells typically exhibit less heterogeneity in nuclear areas and CS values. As with the PEO1/4 cells, the A2780s/cp cells also exhibited similar levels of CIN as demonstrated by largely superimposable cumulative nuclear area distribution frequencies (S7 Fig). Accordingly, unlike the increases in CIN observed within the drug resistant EOC samples (*e*.*g*. EOC13, EOC16 and EOC140), CIN does not increase within the drug resistant cell lines. Thus, our study challenges the suitability of the PEO1/4 and A2780s/cp cell models to accurately reflect the aberrant biology observed in the EOC patient samples, specifically with respect to CIN and drug resistance. While surprising, the markedly similar levels of CIN may simply reflect the artificial selection pressures employed to derive and maintain the cell lines, including 2D growth on plastic and extensive culturing. Consequently, the results of this study caution the use and interpretation of results relating drug resistance and CIN derived from the PEO1/PEO4 and A2780s/A2780cp models, and support the use of primary cultures or the development of more representative cellular models[38].

While many studies have shown genome instability is a common feature of EOC[20–29], none have adequately characterized CIN within patient samples or cell lines, particularly in samples collected over time. Typical studies employ karyotypic analyses, CGH, SNP analysis, and next generation sequencing to identify numerous gene copy number variations, losses of heterozygosity, and/or structural rearrangements[20–29, 39–48]. Although these studies have provided fundamental insight into EOC, they are limited in scope as they only use endpoint analyses (*i*.*e*. a single timepoint), and are heavily influenced by population averaging. In fact, these studies typically only ascribe a single consensus karyotype/genotype, and do not describe the extensive heterogeneity that occurs. To assuage these limitations, we recently developed and validated two quantitative, single cell imaging approaches[31, 32] capable of characterizing the heterogeneous nature of CIN within populations, which we combined for the first time in the current study. Thus, this study is the first to employ quantitative, single cell analyses to characterize CIN and evaluate its temporal dynamics in serial EOC samples. Having observed CIN is dynamic and associated with EOC, we propose CIN is a novel candidate biomarker that may be useful for monitoring disease progression and treatment response. As such, it is tempting to speculate that changes in CIN may help identify and/or distinguish those individuals with responsive or resistant disease, which could conceivably impact clinical management. However, before CIN is established as a novel biomarker in EOC, or any other cancer type, more extensive studies incorporating additional samples are required.

Data gleaned from our study show that CIN is dynamic and increases with drug resistant and recurrent EOCs. These findings are in agreement with a large number of studies in a variety of cancer types that show CIN is associated with the acquisition of drug resistance[16, 18, 35, 49]. Paradoxically, there is a body of evidence that suggests increases in genome instability may also be associated with improved biological fitness and survival outcome[20, 50, 51]. Importantly however, the increases in genome instability often described within these studies does not necessarily specifically relate to CIN, and many researchers frequently use these terms interchangeably despite very different classical definitions. Indeed, CIN is only one of several mechanisms proposed to impact genome stability, which may also include defects in the DNA repair response, microsatellite instability and CpG island methylator phenotype. Further, many of the studies relating CIN to survival outcome typically employ next generation sequencing, SNP analyses or CGH to characterize changes in the abundance of gene copy numbers, SNPs or chromosomal segments as proxies for CIN, but do not actually assess chromosome numbers, particularly at the single cell level. Accordingly, these studies are likely identifying the most frequently occurring driver and passenger alterations that do not necessarily involve whole chromosomes or large parts thereof (*i*.*e*. CIN). While these studies have provided useful insights related to various types of genome instability, they are incapable of characterizing the cell-to-cell heterogeneity in chromosome numbers that is expected to occur in CIN+ (*e*.*g*. CIN containing) tumors. For example, these studies typically employ combined DNA extracted from large cellular populations (*e*.*g*. 10^3^ to 10^6^ cells) and are inherently incapable of detecting or characterizing subtle differences in chromosome numbers predicted to occur in a heterogeneous population of CIN+ tumor cells. Finally, and perhaps most importantly, none of the previous studies have specifically evaluated the temporal kinetics of CIN within a given EOC patient over time or in response to drug treatments, disease recurrence and resistant disease. Thus, the approach employed in the current study provides several novel and key advancements over more traditional approaches.

In general, there is good concordance between the nuclear areas and CS values calculated for each of the EOC patients and the paired cell line models. However, there are instances where the two metrics appear to differ (see EOC140; Fig 5). Although uncommon, this can be expected and likely reflects the inherent differences in the sensitivity of each approach, and is precisely why these approaches have been combined. For example, significant changes in nuclear areas are most likely to reflect large scale changes in DNA content (*i*.*e*. tetraploidy), rather than small scale changes associated with the gain or loss of small subsets of chromosomes (*i*.*e*. 1 or 2 chromosomes). Conceivably, these large scale changes could be envisioned to occur as a result of chromosome segregation defects that may occur following mitotic checkpoint escape, and result in the formation of a tetraploid nucleus. On the other hand, significant differences in CS values are expected to reflect both small and large scale changes in chromosome numbers, provided those changes involve at least one of the three chromosomes evaluated (8, 11, or 17). However, the CS approach is incapable of discerning subtle differences occurring between a tetraploid cell (i.e. 4 copies of all chromosomes; 92 chromosomes) versus an aneuploid cell (52 chromosomes) harboring 4 copies each of chromosomes 8, 11 and 17. Accordingly, the concordance/discordance observed in nuclear areas and CS values for any given sample may simply reflect an underlying difference in the mechanism(s) giving rise to the CIN phenotype. It is for these reasons we believe the concurrent assessment of both nuclear areas and CS values is critical to assemble a more comprehensive assessment of CIN and its dynamics within EOC patient samples.

Finally, although the current approach monitors CIN in EOC cells isolated from ascites, only ~30% of women with EOC produce ascites. To enhance the utility of this approach, we propose it could be modified and applied to assess CIN in both solid and liquid tumour biopsies. In support of this possibility, we previously developed the CS approach and applied it in mini-tissue array comprised of 141 colorectal cancer samples[32]. Thus, we propose the current approach, with slight modifications, could be applied to evaluate CIN in EOC tissues and circulating tumour cells isolated from peripheral blood. As such, nuclear areas and CS values (i.e. CIN analyses) would have broad implications for EOC and virtually any cancer where biopsy or circulating tumour cells are available. However, the general applicability and utility of this approach in monitoring and predicting disease outcome is premature and requires subsequent studies involving appropriate sample sources (*e*.*g*. ascites, biopsy or circulating tumour cells).

## Materials and methods

### Cell culture

Institutional approval for research with human materials was received prior to the initiation of these studies (University of Manitoba Research Ethics Board, #HS19422), and samples were used after receiving informed written consent. Serial ascites obtained by paracentesis from patients with adenocarcinoma was used as a source of primary human EOC cells, and grown as previously described[52]. While numerous primary EOC samples were obtained from patients via paracentesis, some cell samples may not have grown adequately to conduct experiments. All experiments with primary EOC cells were performed between passages 1 and 3. To determine tumor cell purity within the patient samples, quantitative imaging microscopy was performed using a PAX8 antibody (Abcam; ab189249, 1:200), as all tumor cells will be PAX8+ and all potential contaminating normal mesothelial cells are PAX8-. We confirm that >99% of cells are PAX8+ from each of the EOC samples evaluated (>200 cells/sample). PEO1 and PEO4 are cisplatin sensitive and resistant lines[35], respectively, and were purchased from Sigma-Aldrich. PEO cells were grown in RPMI 1640 supplemented with fetal bovine serum (10%), 2 mM glutamine, and 2 mM sodium pyruvate. The cisplatin sensitive human ovarian carcinoma cell line A2780s, and its cisplatin resistant derivative, A2780cp, were obtained from Dr. B. Tsang (University of Ottawa). Short tandem repeat profiling was employed to authenticate the A2780 cells in June 2016 using the Promega PowerPlex system. All cells, with the exception of the PEO1/4 were cultured in DMEM/F12 (HyClone) supplemented with fetal bovine serum (10%). All cells were maintained at 37°C, 5% CO_2_ and 100% humidity.

### FISH

Chromosome enumeration FISH probes (CEPs) specifically recognizing the pericentric regions of chromosomes 8 (CEP8; SpectrumOrange), 11 (CEP11; SpectrumGreen), and 17 (CEP17; Spectrum Aqua) were purchased from Vysis (Abbott Molecular Inc., Mississauga, Ontario, Canada). Chromosomes 8, 11, and 17 were purposefully selected as many genes implicated in ovarian cancer pathogenesis map to these chromosomes including *MYC* (chromosome 8), *CDK12*, and *ATM* (chromosome 11), *TP53*, *BRCA2*, and *NF1* (chromosome 17)[23, 53, 54]. FISH was performed according to the manufacturer (Vysis) with slight modifications. Briefly, cells were seeded into chamber slides ~24 h prior to fixation with 3:1 methanol:acetic acid and pepsin treatment. Cells were rinsed in PBS prior to incubation within a 1×PBS/50mM MgCl_2_ solution. Samples were transferred to a 1% formaldehyde/1×PBS/50mM MgCl_2_ and a 1×PBS solution, and subsequently dehydrated using an ethanol series (70%, 90% and 100%). DNA was denatured with 70% formamide/2×SSC buffer at 70°C for 2 min, dehydrated using an ethanol series (70%, 90% and 100%) and stored at -20°C. The CEP cocktail was prepared by combining CEP11 and CEP17 with the pre-diluted CEP8, and applied to the cells and incubated overnight in a ThermoBrite slide system at 37°C. The following day, samples were washed, counterstained with DAPI, mounted in Vectashield anti-fade reagent (Vector Laboratories) and stored at -20°C until imaged.

### Image acquisition

Two-dimensional images were acquired using a Zeiss AxioImager Z2 microscope equipped with a Plan-Neofluar 20x objective (numerical aperture 0.5) and an AxioCam HR charge-coupled device (CCD) camera. Images were collected using DAPI, CFP (SpectrumAqua), FITC (SpectrumGreen) and Cy3 (SpectrumOrange) filter cubes, and a minimum of 180 nuclei/sample was imaged. All images were imported into Imaris v7.7.2 (Bitplane), where image visualization and analyses were performed (see below).

### Nuclear area analyses

All images were imported into Imaris where nuclear areas were automatically determined as detailed elsewhere[31]. Briefly, size exclusion filters were employed to eliminate apoptotic bodies and overlapping nuclei, while signal intensity thresholds were applied to remove brightly fluorescing apoptotic bodies and mitotic cells. An edge filter was also applied to remove partial nuclei located along the periphery of each image so that only intact interphase nuclei were analyzed.

### CEP analyses

All images were imported into Imaris where the numbers of CEPs within each nucleus were automatically determined as detailed elsewhere[32], but using the same filters/thresholds detailed above (*i*.*e*. size, signal intensity, and edge). All data were exported into Prism v7 (GraphPad) for statistical analyses (see below) and all figures were generated in Photoshop CS6 (Adobe).

### CIN analyses

We previously developed a metric called a CS that describes both the gains and losses of chromosomes (*i*.*e*. CEP foci) so that quantitative comparisons can be made within and between samples[32]. The CS describes the gains and losses of CEP foci within a given nucleus and can be calculated for each individual CEP (*i*.*e*. CS_8_, CS_11_, and CS_17_) or all three CEPs combined (CS_C_). A CEP-specific CS value (*e*.*g*. CS_8_) is calculated by the following formula, [CS_8_ = |*e*_Chr8_—*o*_Chr8_|], where the CS equals the absolute value obtained when the observed (*o*) number of CEP8 foci is subtracted from the expected (*e*) number of two CEP8 foci (expect 1 focus/chromosome × 2 chromosomes/cell), while a CS_C_ value is generated by summing the three CEP-specific CS values (*e*.*g*. CS_C_ = CS_8_ + CS_11_ + CS_17_). By definition, a CS_C_ = 0 identifies a diploid state for the specific chromosomes under investigation. To generate an overall mCS for a given sample, the individual CS values for all the enumerated nuclei in the sample are summed and divided by the total number of nuclei evaluated (n);

$$mCS=\frac{1}{n}\sum_{i=1}^{n}{\left(\left|e_{8}-o_{8}|+|e_{11}-o_{11}|+|e_{17}-o_{17}\right|\right)}_{i}$$

### Statistical analysis

All data were imported into Prism v7 where standard statistics and analyses were generated including mean, standard deviation and Student’s *t*-tests. Differences between the cumulative distribution frequencies for nuclear areas and the CS values (combined [CS_C_] and individual [CS_8_, CS_11_, or CS_17_] and combined) were statistically compared using a two-sample KS-test with a confidence interval of 0.05 (α statistic).

## Supporting information

S1 Fig Primary EOC Samples Can Exhibit Extreme Levels of CIN. **(A)** High resolution image of a nucleus isolated from sample G of EOC16. DNA is counterstained with DAPI (blue), while CEPs 8, 11 and 17 are labeled red, green and yellow, respectively, within the Merge. Presented are the widefield fluorescent images (top) and the corresponding rendered images (bottom). Note the scale bar represents 5 μm and the nucleus is markedly larger than those presented in Fig 1A. The CS_8_ (41), CS_11_ (31) and CS_17_ (21) values are included and collectively produce a CS_C_ value = 93 indicating an extreme level of aneuploidy and CIN within this cell.
(TIF)

S2 Fig CIN is Dynamic and Changes in Response to Carboplatin/Paclitaxel Treatments. **(A)** Cumulative distribution frequency graph presenting the nuclear areas arranged smallest to largest for each sample evaluated from EOC73. **(B)** Graph presenting the cumulative distribution frequencies for CS_C_ values from each sample. **(C)** Cumulative distribution frequency graphs for CS_8_ (left), CS_11_ (middle), and CS_17_ (right).
(TIF)

S3 Fig Increases in CIN Occur in Recurrent EOC. **(A)** Graph presenting the cumulative nuclear area distribution frequencies presented from smallest to largest for each sample evaluated from EOC13. **(B)** Graph presenting the cumulative distribution frequencies for CS_C_ values. **(C)** Cumulative distribution frequency graphs for CS_8_ (left), CS_11_ (middle), and CS_17_ (right).
(TIF)

S4 Fig High Levels of CIN are Associated with Recurrent EOC. **(A)** Graph depicting the cumulative distribution frequency of all nuclear areas (presented smallest to largest) evaluated in samples collected from EOC140. **(B)** Cumulative distribution frequency graph presenting the CS_C_ values from each sample. **(C)** Individual cumulative distribution frequency graphs for CS_8_ (left), CS_11_ (middle) and CS_17_ (right).
(TIF)

S5 Fig CIN Increases in Aggressive, Platinum Resistant EOC. **(A)** Cumulative distribution frequency graph for nuclear areas (presented smallest to largest) evaluated in samples collected from EOC16. **(B)** Cumulative distribution frequency graph presenting the CS_C_ values from each sample. **(C)** Cumulative frequency distribution graphs presenting the individual CS values from each nucleus quantified within each sample.
(TIF)

S6 Fig The Levels of CIN Appear Static in PEO1 and PEO4 Cells. **(A)** Cumulative distribution frequency graph for all nuclear areas measured within PEO1 and PEO4 (presented smallest to largest) indicating the nuclear areas are largely similar in both lines. **(B)** Cumulative distribution frequency graph for CS_C_ values from PEO1 and PEO4 cells. **(C)** Cumulative distribution frequency graphs for CS_8_ (left), CS_11_ (middle) and CS_17_ (right) from PEO1 and PEO4.
(TIF)

S7 Fig A2780s and A2780cp Cells Exhibit Similar Levels of CIN. **(A)** Scatter plot (left) depicting the nuclear area distribution for A2780s (sensitive) and A2780cp (resistant) cells with the interquartile ranges (25^th^, 50^th^ and 75^th^ percentiles) identified in red. Cumulative distribution frequency graph (right) for all nuclear areas measured within A2780s and A2780cp arranged smallest to largest. **(B)** Scatter plot (left) depicting the CS_C_ distribution for nuclei in A2780s and A2780cp cells. Cumulative CS_C_ distribution frequency graph (right) from A2780s and A2780cp cells. **(C)** Scatter plots presenting the gains and losses of CEP 8 (CS_8_; left), 11 (CS_11_; middle) and 17 (CS_17_; right) for each nucleus analyzed in A2780s and A2780cp. **(D)** Cumulative distribution frequency graphs for CS_8_ (left), CS_11_ (middle) and CS_17_ (right) from A2780s and A2780cp.
(TIF)

S1 Table Primary EOC Patient Sample Clinical Details. ^A^optimal surgical debulking (<5 mm)
^B^no surgery
^C^total abdominal hysterectomy, bilateral salpingo-oophorectomy, omentectomy (no note on debulking).
(DOCX)

S2 Table Nuclear Area Statistics for Patient Samples. ^A^Presented in numerical order
^B^Number of nuclei analyzes (N)
^C^Standard deviation (SD)
^D^Fold increase in mean nuclear area relative to the earliest sample collected from a given patient (N/A; not applicable).
(DOCX)

S3 Table KS-tests Comparing the Cumulative Nuclear Area Distribution Frequencies in EOC18^A^. ^A^Presented are the *p*-values calculated from two-sample KS-tests for the indicated pairs with *p*-values <0.05 are considered statistically significant.
(DOCX)

S4 Table KS-tests Comparing the Cumulative CS Distribution Frequencies in EOC18^A^. ^A^Presented are the *p*-values calculated from two-sample KS-tests for the indicated pairs with *p*-values <0.05 are considered statistically significant.
(DOCX)

S5 Table KS-tests Comparing the Cumulative Nuclear Area Distribution Frequencies in EOC73^A^. ^A^Presented are the *p*-values calculated from two-sample KS-tests for the indicated pairs with *p*-values <0.05 considered statistically significant.
(DOCX)

S6 Table KS-tests Comparing the Cumulative CS Distribution Frequencies in EOC73^A^. ^A^Presented are the *p*-values calculated from two-sample KS-tests for the indicated pairs with *p*-values <0.05 considered statistically significant.
(DOCX)

S7 Table KS-tests Comparing the Cumulative Nuclear Area Distribution Frequencies in EOC13^A^. ^A^Presented are the *p*-values calculated from two-sample KS-tests for the indicated pairs with *p*-values <0.05 considered statistically significant.
(DOCX)

S8 Table KS-tests Comparing the Cumulative Nuclear Area Distribution Frequencies in EOC140^A^. ^A^Presented are the *p*-values calculated from two-sample KS-tests for the indicated pairs with *p*-values <0.05 are considered statistically significant.
(DOCX)

S9 Table KS-tests Comparing the Cumulative CS Distribution Frequencies in EOC13^A^. ^A^Presented are the *p*-values calculated from two-sample KS-tests for the indicated pairs with *p*-values <0.05 considered statistically significant.
(DOCX)

S10 Table KS-tests Comparing the Cumulative Nuclear Area Distribution Frequencies in EOC140^A^. ^A^Presented are the *p*-values calculated from two-sample KS-tests for the indicated pairs with *p*-values <0.05 are considered statistically significant.
(DOCX)

S11 Table KS-tests Comparing the Cumulative Nuclear Area Distribution Frequencies in EOC16^A^. ^A^Presented are the *p*-values calculated from two-sample KS-tests for the indicated pairs with *p*-values <0.05 considered statistically significant.
(DOCX)

S12 Table KS-tests Comparing the Cumulative CS Distribution Frequencies in EOC16^A^. ^A^Presented are the *p*-values calculated from two-sample KS-tests for the indicated pairs with *p*-values <0.05 considered statistically significant.
(DOCX)

S13 Table Nuclear Area Statistics for PEO1/4 and A2780s/cp Cells. ^A^Number of nuclei analyzes (N)
^B^Standard deviation (SD)
^C^Fold increase in mean nuclear area.
(DOCX)
