# Supplementary figures and images for: The temporal dynamics of chromosome instability in ovarian cancer cell lines and primary patient samples
Source: PLoS Genet. 2017 Apr 4;13(4):e1006707. doi: 10.1371/journal.pgen.1006707 (PMC5395197; doi:10.1371/journal.pgen.1006707)

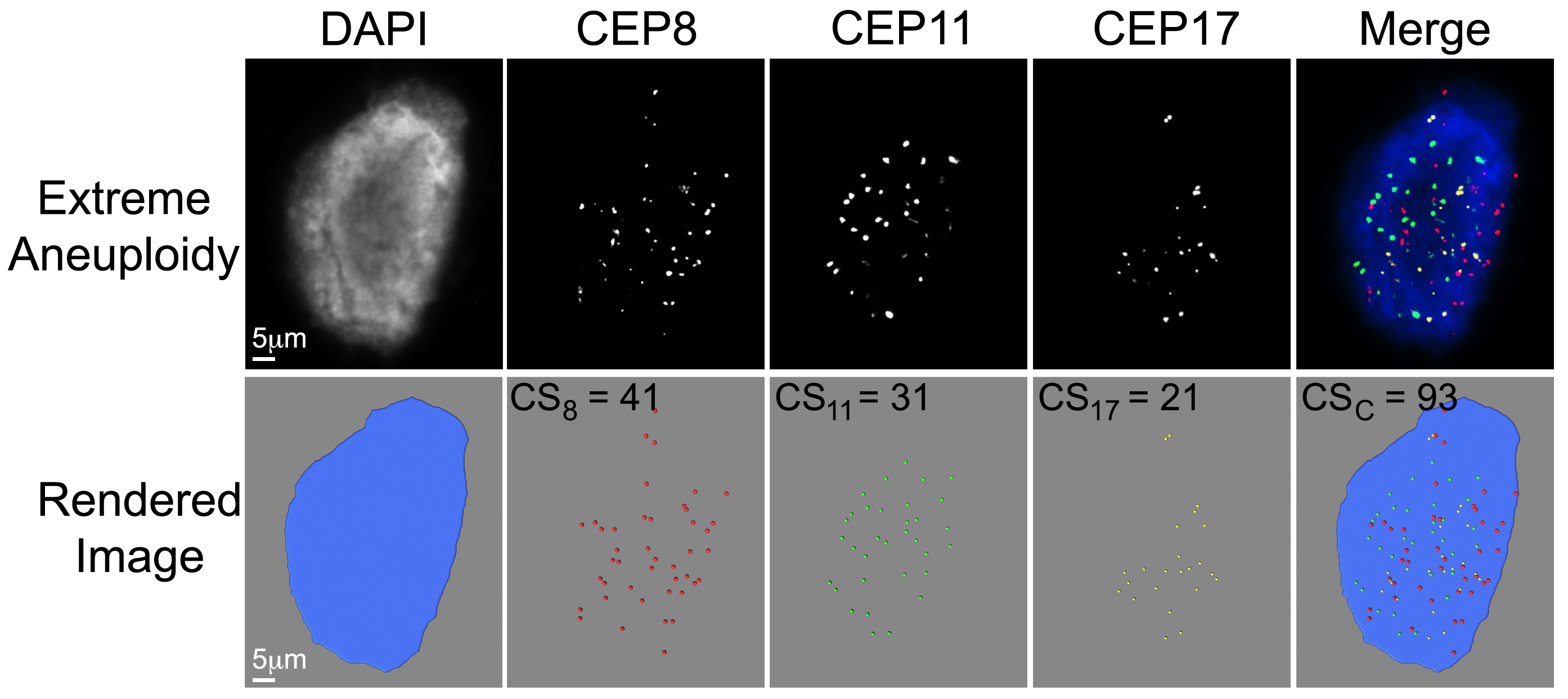

Supplement: S1 Fig — (A) High resolution image of a nucleus isolated from sample G of EOC16. DNA is counterstained with DAPI (blue), while CEPs 8, 11 and 17 are labeled red, green and yellow, respectively, within the Merge. Presented are the widefield fluorescent images (top) and the corresponding rendered images (bottom). Note the scale bar represents 5 μm and the nucleus is markedly larger than those presented in Fig 1A. The CS8 (41), CS11 (31) and CS17 (21) values are included and collectively produce a CSC value = 93 indicating an extreme level of aneuploidy and CIN within this cell. (TIF) [file pgen.1006707.s001.tif]

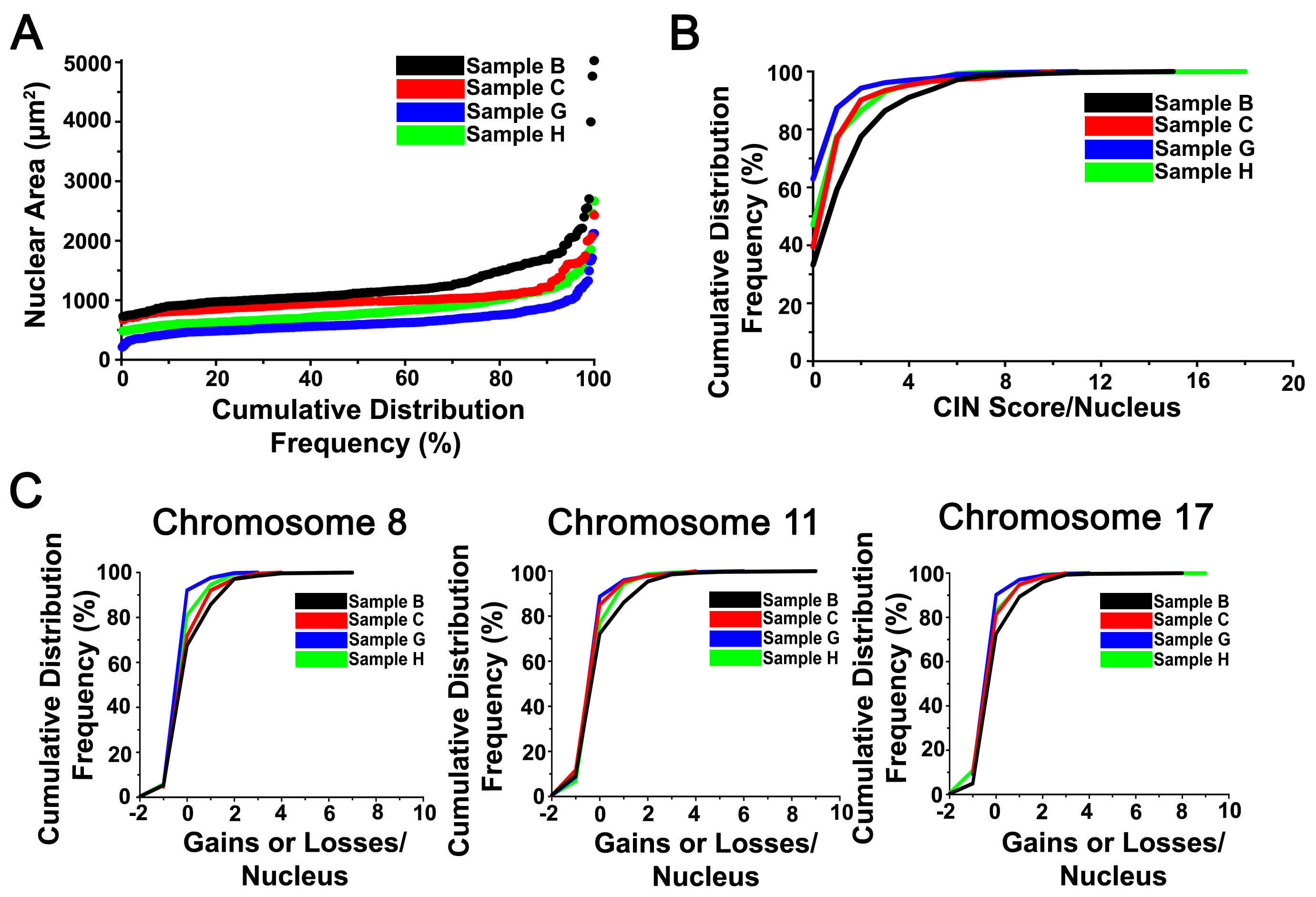

Supplement: S2 Fig — (A) Cumulative distribution frequency graph presenting the nuclear areas arranged smallest to largest for each sample evaluated from EOC73. (B) Graph presenting the cumulative distribution frequencies for CSC values from each sample. (C) Cumulative distribution frequency graphs for CS8 (left), CS11 (middle), and CS17 (right). (TIF) [file pgen.1006707.s002.tif]

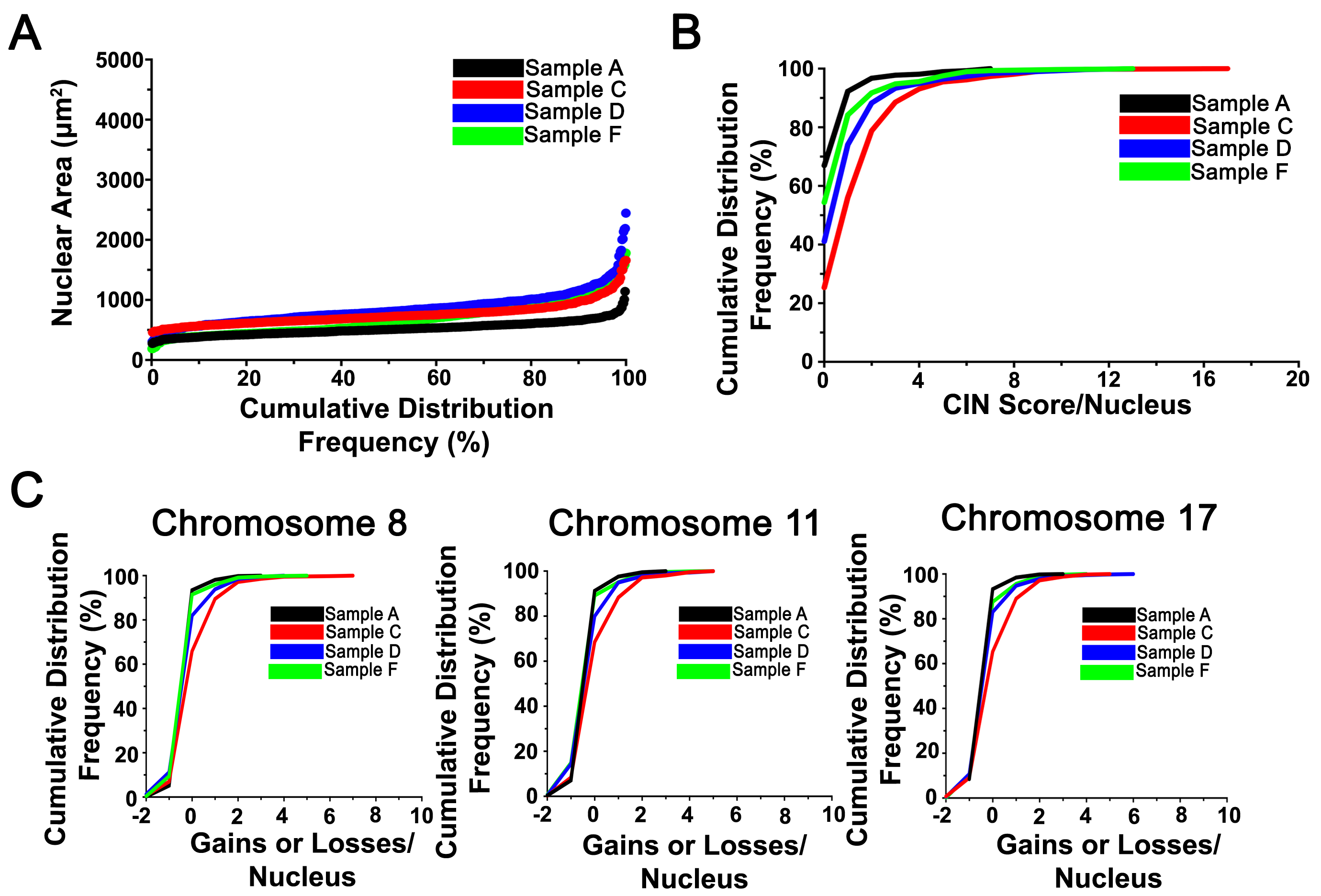

Supplement: S3 Fig — (A) Graph presenting the cumulative nuclear area distribution frequencies presented from smallest to largest for each sample evaluated from EOC13. (B) Graph presenting the cumulative distribution frequencies for CSC values. (C) Cumulative distribution frequency graphs for CS8 (left), CS11 (middle), and CS17 (right). (TIF) [file pgen.1006707.s003.tif]

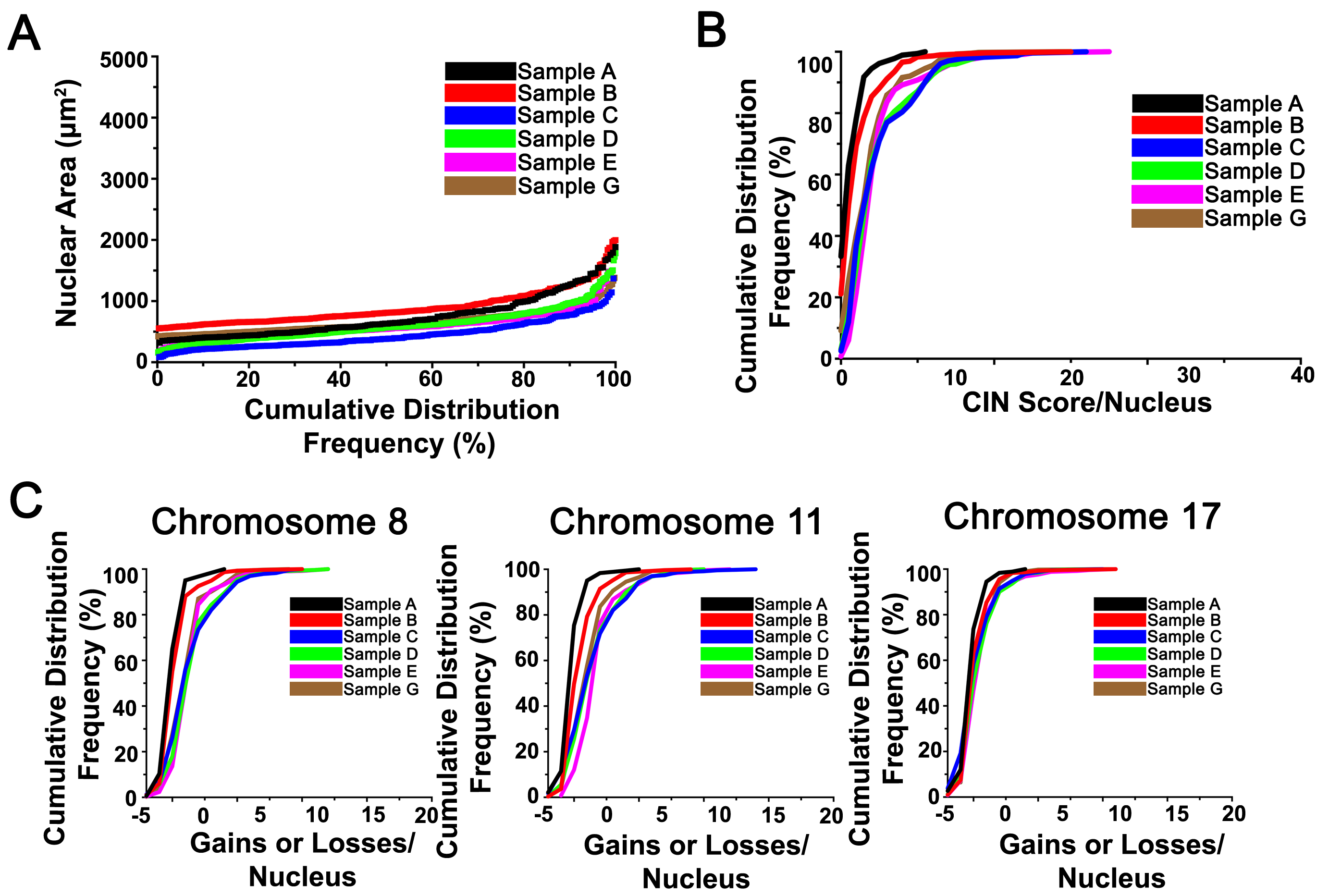

Supplement: S4 Fig — (A) Graph depicting the cumulative distribution frequency of all nuclear areas (presented smallest to largest) evaluated in samples collected from EOC140. (B) Cumulative distribution frequency graph presenting the CSC values from each sample. (C) Individual cumulative distribution frequency graphs for CS8 (left), CS11 (middle) and CS17 (right). (TIF) [file pgen.1006707.s004.tif]

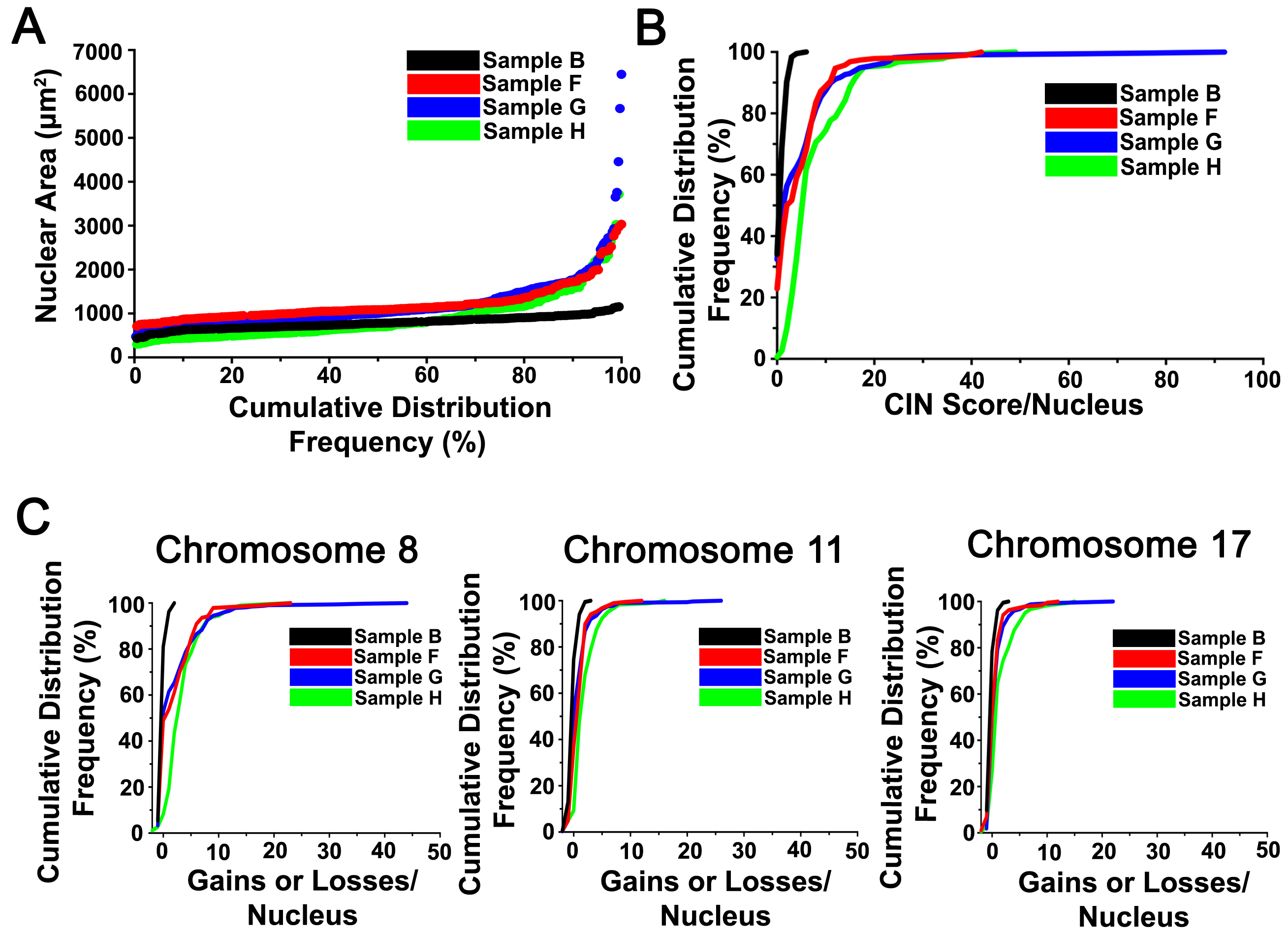

Supplement: S5 Fig — (A) Cumulative distribution frequency graph for nuclear areas (presented smallest to largest) evaluated in samples collected from EOC16. (B) Cumulative distribution frequency graph presenting the CSC values from each sample. (C) Cumulative frequency distribution graphs presenting the individual CS values from each nucleus quantified within each sample. (TIF) [file pgen.1006707.s005.tif]

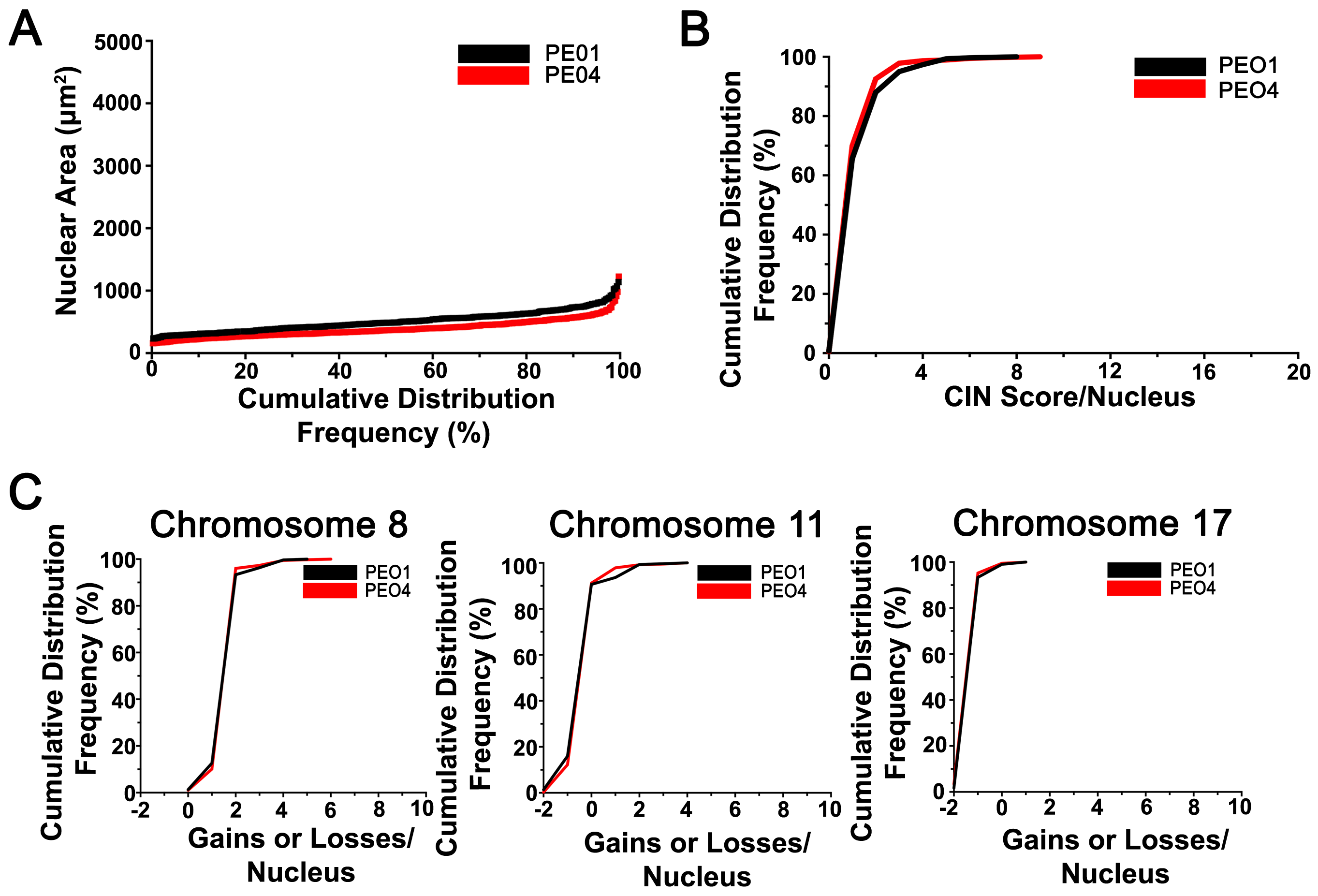

Supplement: S6 Fig — (A) Cumulative distribution frequency graph for all nuclear areas measured within PEO1 and PEO4 (presented smallest to largest) indicating the nuclear areas are largely similar in both lines. (B) Cumulative distribution frequency graph for CSC values from PEO1 and PEO4 cells. (C) Cumulative distribution frequency graphs for CS8 (left), CS11 (middle) and CS17 (right) from PEO1 and PEO4. (TIF) [file pgen.1006707.s006.tif]

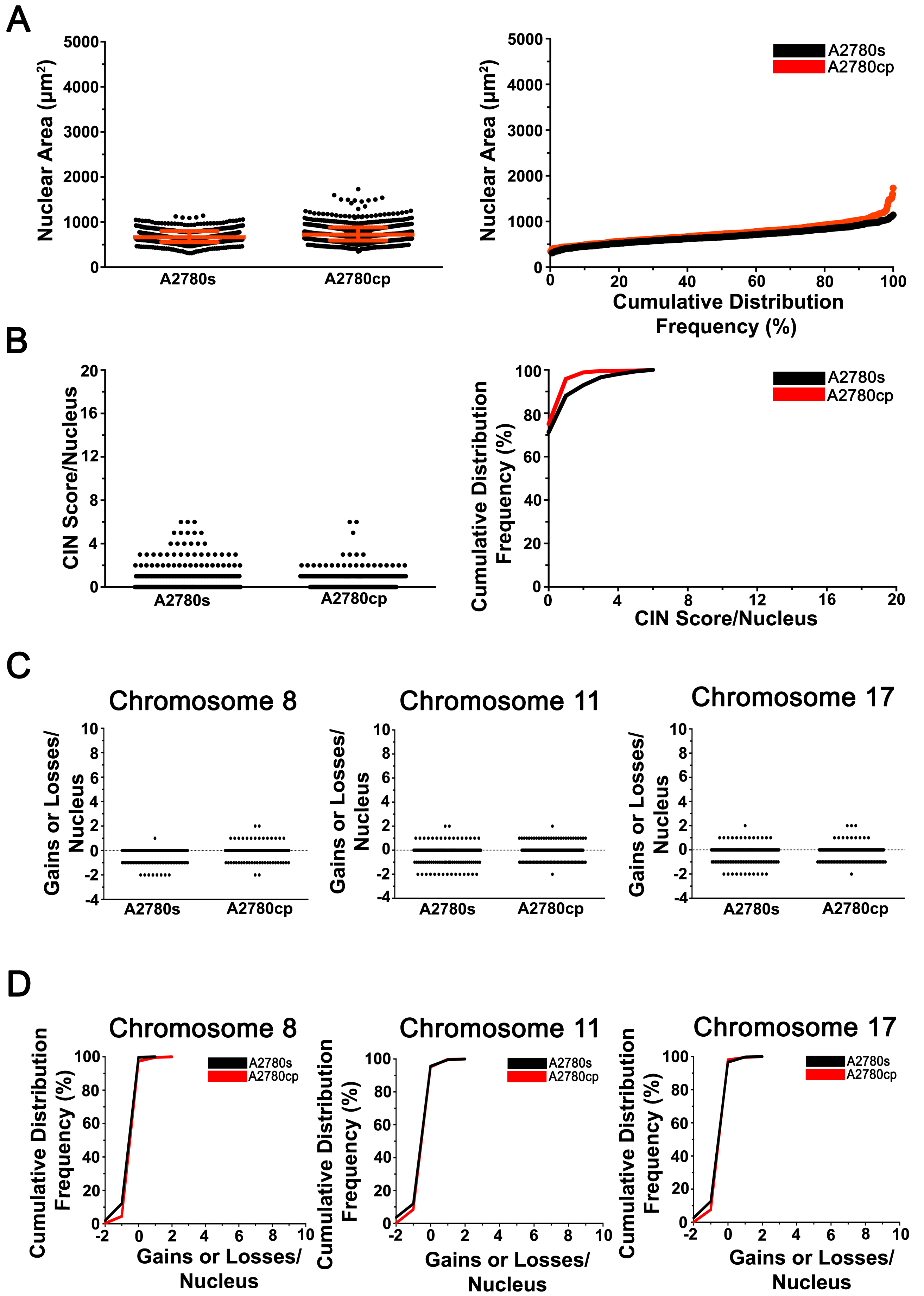

Supplement: S7 Fig — (A) Scatter plot (left) depicting the nuclear area distribution for A2780s (sensitive) and A2780cp (resistant) cells with the interquartile ranges (25th, 50th and 75th percentiles) identified in red. Cumulative distribution frequency graph (right) for all nuclear areas measured within A2780s and A2780cp arranged smallest to largest. (B) Scatter plot (left) depicting the CSC distribution for nuclei in A2780s and A2780cp cells. Cumulative CSC distribution frequency graph (right) from A2780s and A2780cp cells. (C) Scatter plots presenting the gains and losses of CEP 8 (CS8; left), 11 (CS11; middle) and 17 (CS17; right) for each nucleus analyzed in A2780s and A2780cp. (D) Cumulative distribution frequency graphs for CS8 (left), CS11 (middle) and CS17 (right) from A2780s and A2780cp. (TIF) [file pgen.1006707.s007.tif]
