# Supplementary material for: The temporal dynamics of chromosome instability in ovarian cancer cell lines and primary patient samples
Source: PLoS Genet. 2017 Apr 4;13(4):e1006707. doi: 10.1371/journal.pgen.1006707 (PMC5395197; doi:10.1371/journal.pgen.1006707)
Supplement: S1 Table — Aoptimal surgical debulking (<5 mm) Bno surgery Ctotal abdominal hysterectomy, bilateral salpingo-oophorectomy, omentectomy (no note on debulking). (DOCX) [file pgen.1006707.s008.docx]

**S1 Table. Primary EOC Patient Sample Clinical Details**

**Sample Histotype Stage/Grade Comments**

EOC13^A^ Ovarian high grade serous Stage IIIC/High Primary site was right

adenocarcinoma grade fallopian tube

EOC16^B^ Ovarian adenocarcinoma Stage III/High grade Bilateral ovarian tumors

EOC18^B^ Ovarian adenocarcinoma Poor surgical Primary site was right

candidate; patient ovary. Diagnosed via

refused chemotherapy cytology from ascites

advanced stage

EOC73^C^ Ovarian carcinosarcoma Stage IIIC/High grade Epithelial component:

High grade serous

adenocarcinoma

Sarcomatous component:

High grade spindle cell

sarcoma

EOC140^A^ Ovarian high grade serious Stage IIIC/High grade Bilateral ovarian tumours

Cystadenocarcinoma

^A^optimal surgical debulking (<5 mm)

^B^no surgery

^C^total abdominal hysterectomy, bilateral salpingo-oophorectomy, omentectomy (no note on debulking)
