# Supplementary material for: The temporal dynamics of chromosome instability in ovarian cancer cell lines and primary patient samples
Source: PLoS Genet. 2017 Apr 4;13(4):e1006707. doi: 10.1371/journal.pgen.1006707 (PMC5395197; doi:10.1371/journal.pgen.1006707)
Supplement: S2 Table — APresented in numerical order BNumber of nuclei analyzes (N) CStandard deviation (SD) DFold increase in mean nuclear area relative to the earliest sample collected from a given patient (N/A; not applicable). (DOCX) [file pgen.1006707.s009.docx]

**S2 Table. Nuclear Area Statistics for Patient Samples.**

**Nuclear Area (μm^2^)**

**25^th^ 75^th^ Fold**

**Patient^A^ Sample N^B^ Percentile Median Percentile Mean SD^C^ Increase^D^**

EOC13 A 581 438.2 508.0 588.7 520.2 119.7 N/A

C 426 633.8 722.9 817.2 751.8 182.6 1.45

D 614 672.8 807.8 975.1 849.5 267.4 1.63

F 773 457.6 591.6 852.8 676.6 280.5 1.30

EOC16 B 185 684.3 781.1 880.8 786.2 146.3 N/A

F 188 958.3 1087.4 1262.4 1211.0 421.6 1.54

G 327 862.8 1005.3 1383.7 1210.4 639.2 1.54

H 184 520.7 698.3 1114.9 913.0 612.3 1.16

EOC18 B 236 515.9 597.2 702.6 639.7 206.8 N/A

C 262 495.0 592.0 693.5 615.1 182.0 0.96

D 256 488.3 557.7 357.9 584.4 132.3 0.92

E 220 532.0 621.8 736.2 662.6 196.0 1.04

H 300 510.1 588.3 742.3 675.8 323.9 1.06

I 368 661.2 776.2 999.1 865.7 297.2 1.35

EOC140 A 183 469.9 632.6 882.4 737.8 345.2 N/A

B 294 675.3 810.6 1021.0 886.1 282.5 1.20

C 234 271.2 384.2 577.3 447.5 236.4 0.61

D 504 414.3 570.8 745.2 608.2 275.5 0.82

E 250 440.6 346.3 697.3 597.7 260.2 0.81

G 404 517.8 604.9 753.3 658.8 194.1 1.12

EOC73 B 281 992.1 1118.9 1372.0 1250.5 493.9 N/A

C 213 844.7 976.8 1048.6 1016.6 248.8 0.81

G 469 503.1 590.8 714.8 636.9 226.8 0.51

H 295 653.6 771.7 945.6 845.5 292.3 0.68

^A^Presented in numerical order

^B^Number of nuclei analyzes (N)

^C^Standard deviation (SD)

^D^Fold increase in mean nuclear area relative to the earliest sample collected from a given patient (N/A; not applicable)
