# Supplementary material for: The temporal dynamics of chromosome instability in ovarian cancer cell lines and primary patient samples
Source: PLoS Genet. 2017 Apr 4;13(4):e1006707. doi: 10.1371/journal.pgen.1006707 (PMC5395197; doi:10.1371/journal.pgen.1006707)
Supplement: S4 Table — APresented are the p-values calculated from two-sample KS-tests for the indicated pairs with p-values <0.05 are considered statistically significant. (DOCX) [file pgen.1006707.s011.docx]

**S4 Table. KS-tests Comparing the Cumulative CS Distribution Frequencies in EOC18. ^A^**

**Category Sample C D E H I**

CS_C_ B 0.9713 0.9964 0.3936 0.4904 0.0009

C N/A 0.9998 0.9849 0.6355 0.0202

D N/A 0.7948 0.3435 0.0053

E N/A 0.1227 0.0375

H N/A 0.0001

CS_8_ B >0.9999 >0.9999 0.9285 0.3429 0.0105

C N/A >0.9999 0.8921 0.6887 0.0378

D N/A >0.9999 0.7311 0.0203

E N/A 0.6772 0.1827

H N/A 0.0044

CS_11_ B 0.7883 0.8847 0.9195 0.2051 0.0005

C N/A >0.9999 >0.9999 0.0315 0.0440

D N/A >0.9999 0.1066 0.0149

E N/A 0.1920 <0.0001

H N/A 0.0139

CS_17_ B 0.9993 >0.9999 >0.9999 0.4914 0.0055

C N/A 0.9996 >0.9999 0.8630 0.0278

D N/A >0.9999 0.6924 0.0055

E N/A 0.9331 0.0353

H N/A 0.1513

^A^Presented are the *p*-values calculated from two-sample KS-tests for the indicated pairs with *p*-values <0.05 are considered statistically significant.
