# Supplementary material for: The temporal dynamics of chromosome instability in ovarian cancer cell lines and primary patient samples
Source: PLoS Genet. 2017 Apr 4;13(4):e1006707. doi: 10.1371/journal.pgen.1006707 (PMC5395197; doi:10.1371/journal.pgen.1006707)
Supplement: S5 Table — APresented are the p-values calculated from two-sample KS-tests for the indicated pairs with p-values <0.05 considered statistically significant. (DOCX) [file pgen.1006707.s012.docx]

**S5 Table. KS-tests Comparing the Cumulative Nuclear Area Distribution Frequencies in EOC73.^A^**

**Sample C G H**

B <0.0001 <0.0001 <0.0001

C N/A <0.0001 <0.0001

G N/A <0.0001

^A^Presented are the *p*-values calculated from two-sample KS-tests for the indicated pairs with *p*-values <0.05 considered statistically significant.
