# Supplementary material for: The temporal dynamics of chromosome instability in ovarian cancer cell lines and primary patient samples
Source: PLoS Genet. 2017 Apr 4;13(4):e1006707. doi: 10.1371/journal.pgen.1006707 (PMC5395197; doi:10.1371/journal.pgen.1006707)
Supplement: S6 Table — APresented are the p-values calculated from two-sample KS-tests for the indicated pairs with p-values <0.05 considered statistically significant. (DOCX) [file pgen.1006707.s013.docx]

**S6 Table. KS-tests Comparing the Cumulative CS Distribution Frequencies in EOC73.^A^**

**Category Sample C G H**

CS_C_ B 0.0010 <0.0001 0.0001

C N/A <0.0001 0.4587

G N/A 0.0002

CS_8_ B 0.7305 <0.0001 0.0114

C N/A <0.0001 0.2475

G N/A 0.0232

CS_11_ B 0.0393 0.0001 0.2993

C N/A 0.9872 0.4028

G N/A 0.0135

CS_17_ B 0.3286 <0.0001 0.1053

C N/A 0.1891 >0.9999

G N/A 0.2630

^A^Presented are the *p*-values calculated from two-sample KS-tests for the indicated pairs with *p*-values <0.05 considered statistically significant.
