# Supplementary material for: The temporal dynamics of chromosome instability in ovarian cancer cell lines and primary patient samples
Source: PLoS Genet. 2017 Apr 4;13(4):e1006707. doi: 10.1371/journal.pgen.1006707 (PMC5395197; doi:10.1371/journal.pgen.1006707)
Supplement: S9 Table — APresented are the p-values calculated from two-sample KS-tests for the indicated pairs with p-values <0.05 considered statistically significant. (DOCX) [file pgen.1006707.s016.docx]

**S9 Table. KS-tests Comparing the Cumulative CS Distribution Frequencies in EOC13.^A^**

**Category Sample C D F**

CS_C_ A <0.0001 <0.0001 <0.0001

C N/A <0.0001 <0.0001

D N/A <0.0001

CS_8_ A <0.0001 0.0007 0.5778

C N/A <0.0001 <0.0001

D N/A 0.0039

CS_11_ A <0.0001 0.0010 0.0346

C N/A 0.0028 <0.0001

D N/A 0.0064

CS_17_ A <0.0001 0.0039 0.2302

C N/A <0.0001 <0.0001

D N/A 0.4869

^A^Presented are the *p*-values calculated from two-sample KS-tests for the indicated pairs with *p*-values <0.05 considered statistically significant.
