# Supplementary material for: The temporal dynamics of chromosome instability in ovarian cancer cell lines and primary patient samples
Source: PLoS Genet. 2017 Apr 4;13(4):e1006707. doi: 10.1371/journal.pgen.1006707 (PMC5395197; doi:10.1371/journal.pgen.1006707)
Supplement: S10 Table — APresented are the p-values calculated from two-sample KS-tests for the indicated pairs with p-values <0.05 are considered statistically significant. (DOCX) [file pgen.1006707.s017.docx]

**S10 Table. KS-tests Comparing the Cumulative Nuclear Area Distribution Frequencies in EOC140.^A^**

**Category Sample B C D E G**

CS_C_ A 0.0385 <0.0001 <0.0001 <0.0001 <0.0001

B N/A <0.0001 <0.0001 <0.0001 <0.0001

C N/A 0.4524 0.0078 0.0107

D N/A 0.1933 0.0091

E N/A <0.0001

CS_8_ A 0.2165 <0.0001 <0.0001 <0.0001 <0.0001

B N/A <0.0001 <0.0001 <0.0001 <0.0001

C N/A 0.2315 0.0467 0.0082

D N/A 0.2872 0.0165

E N/A 0.0042

CS_11_ A <0.0001 <0.0001 <0.0001 <0.0001 <0.0001

B N/A <0.0001 <0.0001 <0.0001 <0.0001

C N/A 0.6165 0.0005 0.0308

D N/A <0.0001 0.0149

E N/A <0.0001

CS_17_ A 0.1658 0.0146 <0.0001 <0.0001 <0.0001

B N/A 0.0548 0.0106 0.0013 0.0896

C N/A 0.2685 0.0605 0.0156

D N/A 0.8731 0.6611

E N/A 0.4516

^A^Presented are the *p*-values calculated from two-sample KS-tests for the indicated pairs with *p*-values <0.05 are considered statistically significant.
