# Supplementary material for: The temporal dynamics of chromosome instability in ovarian cancer cell lines and primary patient samples
Source: PLoS Genet. 2017 Apr 4;13(4):e1006707. doi: 10.1371/journal.pgen.1006707 (PMC5395197; doi:10.1371/journal.pgen.1006707)
Supplement: S13 Table — ANumber of nuclei analyzes (N) BStandard deviation (SD) CFold increase in mean nuclear area. (DOCX) [file pgen.1006707.s020.docx]

**S13 Table. Nuclear Area Statistics for PEO1/4 and A2780s/cp Cells.**

**Nuclear Area (μm^2^)**

**25^th^ 75^th^ Fold**

**Cell Line N^A^ Percentile Median Percentile Mean SD^B^ Increase^C^**

PEO1 300 380.6 485.9 605.3 510.0 117.1 N/A

PEO4 376 290.4 367.7 465.8 390.9 155.0 0.77

A2780s 411 553.3 661.7 799.4 678.9 174.7 N/A

A2780cp 605 592.4 723.9 881.0 749.0 219.3 1.10

^A^Number of nuclei analyzes (N)

^B^Standard deviation (SD)

^C^Fold increase in mean nuclear area
